# Effect of iron saturation of bovine lactoferrin on the inhibition of hepatitis B virus *in vitro*

**DOI:** 10.7717/peerj.17302

**Published:** 2024-05-07

**Authors:** Haibo Zhou, Yiwei Zhu, Ning Liu, Wencui Zhang, Jianchun Han

**Affiliations:** 1 College of Food Science, Northeast Agricultural University, Harbin, Heilongjiang Province, China; 2 Chongqing Food Industry Research Institute Co., Ltd., Chongqing, China; 3 Institute of Endemic Diseases, Harbin Center for Disease Control and Prevention, Harbin, Heilongjiang Province, China

**Keywords:** Bovine lactoferrin, Hepatitis B virus, Iron saturation, HepG2 cell, Polymerase chain reaction

## Abstract

**Background:**

Hepatitis B virus (HBV) infection poses a major public health problem worldwide. Bovine lactoferrin (bLf) is a natural product that can inhibit HBV, but the effect of iron saturation on its resistance to HBV is unknown.

**Aims:**

The purpose of this study is to investigate the impact of iron saturation of bLf against HBV.

**Methods:**

HepG2 cells were cultured in DMEM high glucose containing 10% inactivated fetal calf serum, at 37 °C, in 5% CO_2_. MTT method was used to detect the cytotoxicity of bLf to HepG2 cells. Apo-bLf and holo-bLf were prepared from bLf. Iron saturation of these proteins was determined by atomic absorption spectrophotometry. Non-cytotoxic concentrations of candidate proteins were used in anti-HBV tests. Fluorescent quantitative polymerase chain reaction was used to detect HBV-DNA.

**Results:**

The TC_50_ and TC_0_of bLf were 54.570 mg/ml and 1.997 mg/ml, respectively. The iron saturation of bLf, apo-bLf and holo-bLf were 10.29%, 8.42% and 85.32%, respectively. In this study, four non-cytotoxic concentrations of candidate proteins (1.5, 1.0, 0.5, and 0.1 mg/ml, respectively) were used to inhibit HBV in HepG2 cells. The results showed that 1.5 mg/ml bLf and 0.1 mg/ml holo-bLf effectively impaired the HBV-DNA amplification in HBV-infected HepG2 cells (*P* < 0.05). However, apo-bLf, and Fe^3+^ did not show the anti-HBV effects.

**Conclusion:**

A total of 1.5 mg/ml bLf and 0.1 mg/ml holo-bLf could inhibit HBV-DNA in HepG2 cells. Complete bLf structure, appropriate concentration and iron saturation of bLf are necessary conditions for anti-HBV effects.

## Introduction

Hepatitis B virus (HBV) infection is one of the major public health problems worldwide. Approximately 3.5% of the global population is chronically infected with HBV and 887,000 people die every year due to the consequences of hepatitis B (WHO, 2017). Although the incidence of HBV infections is decreasing owing to vaccination, the use of antiviral therapy also to some extent reduces the viral load of chronically infected individuals. Nucleotide analogs and interferon (IFN), such as adefovir, lamivudine and entecavir are regular medicines utilized for the treatment of HBV infection. However, the increasingly accumulated drug resistance, relative side effects and low cure rates are the obstacles to application of these medicines (Ascione et al., 2006; Dienstag et al., 1999; Lai et al., 1998). The unsatisfactory therapeutic effect of nucleotide analogs and IFN has strengthened the requirement for new anti-HBV agents.

Lactoferrin (Lf) is a Fe^3+^-binding glycoprotein with a molecular weight of 78 kDa. It was discovered in human epithelial secretions and bovine milk (Baker et al., 2000). There are two lobes in a single polypeptide chain of Lf. Each lobe contains one glycan chain and one ferric ion (Hao et al., 2019). Native lactoferrin is 10–20% iron saturated (Ianiro et al., 2023). Bovine lactoferrin (bLf) and human lactoferrin (hLf) share approximately 70% sequence identity (Yount et al., 2007). The biological activity of bovine lactoferrin have been widely studied. Many studies have reported that lactoferrin can interfere with infection of Japanese encephalitis virus, human immunodeficiency virus-1, herpes simplex virus-1, poliovirus, papillomavirus and mayaro virus (Carvalho et al., 2014; Chien et al., 2008; Marr et al., 2009; McCann et al., 2003; Mistry et al., 2007; Puddu et al., 1998). Hara et al. (2002) reported that bLf or hLf preincubation with PH5CH8 cells could prevent HBV infection. The results of our previous studies show that Zn^2+^-bLf and Fe^2+^-bLf can effectively inhibit the amplification of HBV-DNA in HepG2 cells (Li et al., 2009). This evidence suggests that bLf is candidate and potent inhibitor for effectively inhibit HBV.

As an exceedingly multi-biological function protein, many biologically important functions of bLf are performed by iron binding ability (Baker & Baker, 2012). BLf could have different iron saturation depending on the iron release and binding. In general, two domains of each lobe of Lf close over when binding metal ion, and the domains open when Lf releases iron (Anderson et al., 1990; Gerstein et al., 1993). Two forms of bovine lactoferrin: metal-depleted (apo-bLf) and iron-saturated bLf (holo-bLf) were the most common patterns in this large-scale conformational change. In Puddu et al. (1998) study, apo-bLf and holo-bLf significantly suppressed HIV-1 replication and syncytium formation and curtailed HIV-1 DNA in the C8166 T cell line when added prior to HIV infection or during the viral adsorption step. Similarly, another study revealed that apo-bLf, native bLf, or holo-bLf inhibited viral replication throughout the poliovirus infection cycle or during viral adsorption, but could not suppress an infection stage after viral internalization into the host cells (Marchetti et al., 1999). An earlier study showed that apo-bLf and holo-bLf are able to prohibit attacking HT-29 cell from rotavirus in a dose-dependent manner (Superti et al., 1997). It was shown that apo-lactoferrin hinders virus attachment to cell receptors since it is able to bind the viral particles and to prevent both rotavirus haemagglutination and viral binding to susceptible cells. All aforementioned lactoferrins acted on an early phase of virus infection. At present, it is not clear whether bLf is able to inhibit the infection of HBV after viral internalization into the host cells by a dose-dependent manner of iron saturation. Therefore, the effect of bLf with different iron saturation on HBV was investigated in this study in order to find a more ideal natural protein which could be anti-HBV.

## Materials and Methods

### Chemicals and viruses

BLf was produced by Westland Milk Products company with purity >90%. Iron saturation of BLf (mg iron/100 g of bovine lactoferrin) was detected by atomic absorption spectrophotometry (AA800; PerkinElmer, Waltham, MA, USA). According to GB/T5009.90-2003 standard, FeSO_4_ was dissolved in deionized water and prepared into 0 mg/L, 1 mg/L, 2 mg/L, 3 mg/L, 4 mg/L, 5 mg/L, respectively. A total of 2 mg/ml of protein concentration was added in the mixed acid digestion solution (nitric acid: perchloric acid = 4: 1). After digestion, it was atomized by atomic absorption flame and the absorbance value was determined at 248.3 nm. In a certain concentration range, the absorbance value of iron is directly proportional to the iron content, which is quantitative compared with the standard series. Recombinant human interferon-alpha 2B for injection (IFN-alpha 2B) was purchased from Shenzhen Neptunus Interlong Bio-tech Co., Ltd. Fetal calf serum was obtained from Zhejiang Tianhang Biotechnology Co., Ltd. (Tongxiang, China). DMEM high glucose was afforded by Thermo Fischer Scientific Co., Ltd. (Waltham, MA, USA). Benzylpenicillin sodium for injection and streptomycin sulfate for injection were purchased from Shandong Lukang Pharmaceutical Co., Ltd. (Jining, China). Hepatitis B virus positive serum was collected from 106 patients with hepatitis B (negative for hepatitis A and hepatitis C) in the First Affiliated Hospital of Harbin Medical University. We mixed the serum of 106 patients, and filtered it with a 0.22 nm filter membrane in the biosafety cabinet to remove bacteria. The hepatitis B virus was detected by the First Affiliated Hospital of Harbin Medical University, and its load was 5 ×10^8^ copy/ml. This study was approved by the Ethics Committee of First Afiliated Hospital of Harbin Medical University (IRB-AF/SC-04/02.0). All participants included in this study signed informed consent.

### Cell culture

HepG2 cells were donated from Harbin Medical University, and were cultured in DMEM high glucose containing 10% inactivated fetal calf serum, 100 IU/ml penicillin and 100IU/ml streptomycin at 37 °C, in 5% CO_2_. The media were freshened in the following day (5% inactivated fetal calf serum; for cell maintenance the serum concentration was lowered to 3%) and the cells were passaged every three days.

### Cytotoxicity assay

MTT method was used to detect the cytotoxicity of 24, 12, 6, 3, 1.5 mg/ml of bLf to HepG2 cells by calculating the survival rate of the cells. The MTT (purchased from Labgic Technology, Beijing, China) was dissolved in sterilized phosphate-buffered saline. The final concentration was 5 mg/ml. In each well of 96-Well Cell Culture Plates, 200 ml of 5 × 10^4^/ml HepG2 cells were plated and cultured at 37 °C for 48 h. The reagents above were all dissolved in DMEM high glucose and inoculated to cells with 10% inactivated fetal calf serum, 100 IU/ml penicillin and 100 IU/ml streptomycin at 37 °C, in 5% CO_2_. Untreated cells with equivalent amount of solvent were invoked as control. After 48 h, cytotoxicity assay for bLf effect on HepG2 cells were initiated. The absorbance at wavelength of 550 nm was measured by Microplate Reader (Bio-Rad, Hercules, CA, USA).

### The preparation of apo-bLf and holo-bLf

Apo-bLf was prepared by dialysis against 20 volumes of 0.1 mol/L citric acid (renewed once). After 36 h, the citric acid was eliminated by dialysis against 20 volumes of deionized water (twice renewed) for 24 h at 4 °C. After such treatment, the solution of bLf became completely colorless (Masson & Heremans, 1968). In order to obtain holo-bLf, apo-bLf was dissolved in 0.1 mol/L sodium bicarbonate with 20 µg/ml of ferric chloride at room temperature. After 24 h incubation, the protein was dialysis against 0.1 mol/L NaHCO_2_ to remove unconnected metal ions, and then the holo-bLf was freeze-dried (Marchetti et al., 1999). Spectrofluorophotometer (RF-5301pc; Shimadzu, Kyoto, Japan) was used to prove the bLf structure remained after Fe^3+^ was exhausted or added. During the measurement, 0.03 mg/ml protein was added in one cm cuvette, the width of the fluorescence emission and excitation slit is 5 nm, the excitation wavelength is 285 nm, and the emission spectrum of 290–550 nm is drawn at room temperature. Iron saturation of bLf were determined by atomic absorption spectrophotometry. The protein integrity was detected by SDS-PAGE. The protein concentration was 0.1 mg/ml and the volume is 12 µl. Nanodrop (2000c: Thermo Fisher Scientific, Waltham, MA, USA) was used to detect the concentration of protein solution in each step at the wavelength of 280 nm.

### Infection of HBV to cells

HepG2 cells were seeded on plastic wells at a density of 5 × 10^4^ cells per well in 24-well plates 24 h prior to viral inoculation, each well 1 ml. 100 µl serum which containing 5 × 10^6^ copies/ml HBV was added to 1,000 µl DMEM medium containing HepG2 cells. Then the pH value was adjusted to 5.5. After cultured for 4 h at 37 °C, 1 ml of phosphate-buffered saline (PBS, pH 7.2) was added to wash off the free HBV, this step repeated three times, 0.22 µm filter unit was adopted to filter HBV serum (Gripon et al., 2002; Mabit et al., 1994).

### Anti-HBV effect of lactoferrin with different iron saturation levels

The cells infected by HBV were eluted by PBS and cultured for 48 h, and then the supernatant was removed. A total of 1 ml of PBS was added to wash the cells three times. To evaluate the effect of protein iron saturation on the anti-HBV effect of bLf. Anti-HBV experiments were carried out with apo-bLf, bLf and holo-bLf, respectively. 0.2 ml of candidate protein of each concentration were applied to infected cells. The non-toxic concentrations of each protein are 1.5, 1.0, 0.5 and 0.1 mg/ml, respectively. The infected cells without any treatment are defined as positive groups. 2,500 IU/ml IFN-alpha 2B was selected as the control group of conventional drugs (Zhou et al., 2008). We chose 6 µg/ml FeCl_3_ (equivalent to the iron content of 1.5 mg/ml bLf), which is much less toxic to cells, to be used in antiviral anti-HBV experiments (Terpilowska & Siwicki, 2017). The antiviral time of the above reagents lasted for 48 h. In this study, all sampling was done in triplicate. All reagents are prepared or used after filtering by 0.22 µm filtration membranes to ensure sterility.

### Fluorescence quantitative-polymerase chain reaction (FQ-PCR)

According to the manufacturer’s protocols, DNA extraction of each sample was performed using a DNA Vzol Kit (Vigorous Biotechnology Company, Haidian, China). At 4 °C, all DNA was dissolved in 100 µl TE solution. 2 µl of sample solution measuring was determined. An HBV FQ-PCR diagnostic Kit was utilized to detect the HBV (DaAn Gene Co., Ltd. of Sun Yat-Sen University) on the ABI7500 instrument platform (Applied Biosystems, Waltham, MA, USA). HBV-DNA copies of samples was absolute quantitative analyzed using HBV positive quantitative control standard solution. 1 ×10^8^ copies/ml positive HBV was diluted to five gradients of concentration of tenfold to standard curve formation.

### Statistical analysis

Data points were obtained from at least three different cell cultures, in which each condition was assayed in triplicate. Values were expressed as mean ± SD. To calculate the statistical significance of differences within groups, the one-way ANOVA analysis was utilized. The data is tested for normality. Due to the non-normal distribution of data (Tables S1–S2), we used the Games-Howell test to achieve multiple comparisons between groups under the condition of uneven variance. All the statistical analyses were finished by SPSS 23.0 software. The inspection level (*α*) of bilateral probabilistic tests was 0.05.

## Results

### Preparation of protein and its integrity test

**Figure 1:**
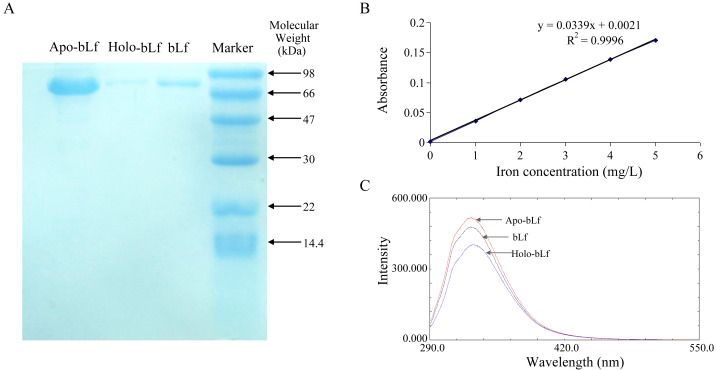
(A) Electrophoretogram of lactoferrin with different iron saturation. (B) The standard curve of iron saturation of lactoferrin. (C) Fluorescence spectra of lactoferrin with different iron saturation.

In this study, survival rate of 6, 3, 1.5 mg/ml of the bLf was not significantly different from normal control (*P* > 0.05, *n* = 4) (Table 1). The result of probit regression analysis showed that the TC_50_ of bLf was 54.570 mg/ml, and TC_0_ = 1.997 mg/ml. To be conservative, 1.5 mg/ml of bLf was selected as the maximum concentration in the subsequent experiment. As shown in Fig. 1A, three candidate proteins integrity was determined using SDS-PAGE stained with coomassie brilliant blue. Electrophoresis showed that the proteins had no tail and other bands, indicating that the protein was not degraded and the purity was good. Atomic absorption spectrophotometry was applied to detect the saturation of iron in bLf. The iron saturation of bLf was 10.29% (mg iron/100 g of bLf), and the iron saturation of apo-bLf and holo-bLf were 8.42% and 85.32%, respectively. The standard curve of iron saturation was shown in Fig. 1B. As shown in Fig. 1C, the results of fluorescence spectrum show that the peak shape of apo-bLf at 336 nm is the highest, followed by bLf, and the lowest is holo-bLf. The fluorescence intensity of apo-bLf is the largest, followed by bLf and holo-bLf, which shows that the combination with iron increases the fluorescence quenching degree of bLf, and the maximum fluorescence intensity of the three proteins is maintained at the wavelength of 336 nm, indicating that iron is a small molecule quencher interacting with bLf.

**Table 1:** Cytotoxicity test of lactoferrin on HepG2 cells *in vitro* (*n* = 4).

bLf (mg/ml)	$\overline{x}$ ± s	Survival rate (%)	*P*
24	*1.293*± *0.036*	70.573	<0.001
12	*1.577*± *0.047*	86.030	<0.001
6	*1.770*± *0.057*	96.603	0.069
3	*1.756*± *0.037*	95.825	0.029
1.5	*1.827*± *0.063*	99.686	0.860
Normal control	*1.832*± *0.018*	–	

### The antiviral effect of candidate protein

**Figure 2:**
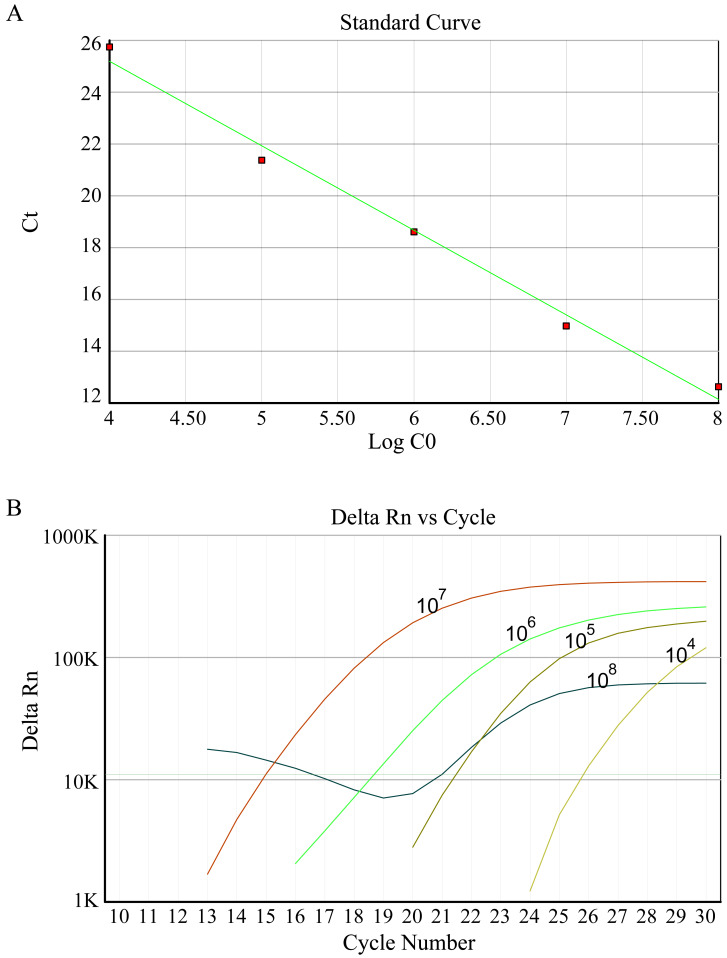
(A) Standard curve of HBV DNA detected by FQ-PCR. (B) FQ-PCR amplification curves of standard, the concentrations of standards are 1 ×10^8^ copies/ml, 1 ×10^7^ copies/ml, 1 ×10^6^ copies/ml, 1 ×10^5^ copies/ml, 1 ×10^4^ copies/ml, respectively.

**Figure 3:**
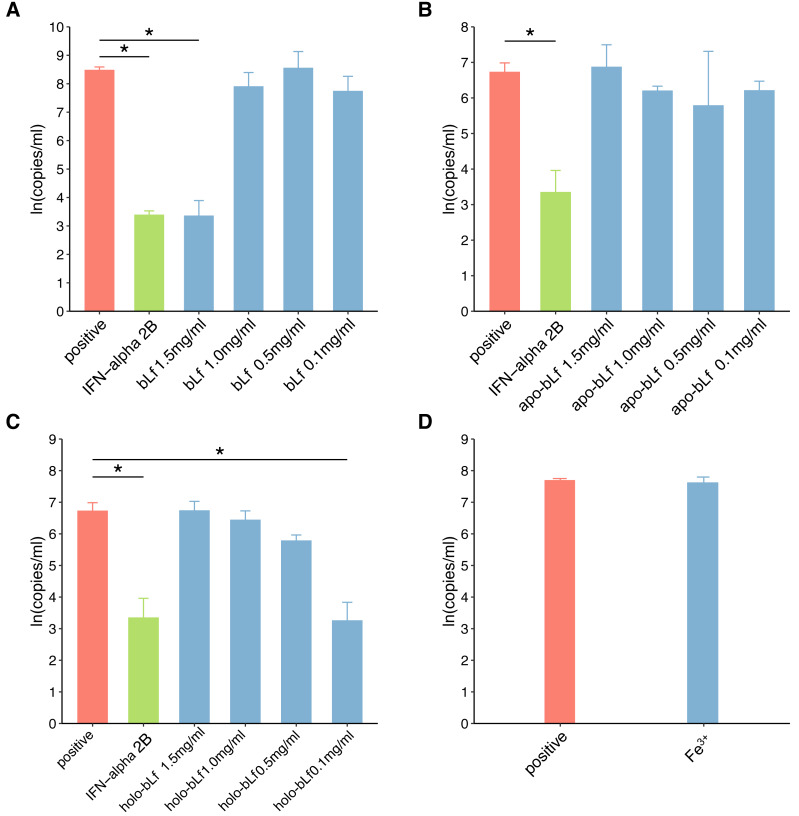
The effect of candidate agents inhibits HBV. Non-toxic concentrations of each protein are 1.5, 1.0, 0.5 and 0.1 mg/ml, respectively. 2,500 IU/ml IFN-alpha and 6 *μ*g/ml FeCl_3_ were involved in this test. The infected cells without any treatment are defined as positive groups. *Y*-axis represents the natural logarithm of HBV copies/ml. (A) Inhibition of bLf on HBV virus. (B) Inhibition of apo-bLf on HBV virus. (C) Inhibition of holo-bLf on HBV virus. (D) Inhibition of Fe^3+^ on HBV virus.

In this study, fluorescence quantitative PCR was used to detect the anti-HBV effect of the candidate protein. Figure 2 shows the standard PCR amplification curve and amplification curve of sample. As shown in Fig. 3, four different concentrations of bLf were used in the anti-HBV test. 1.5 mg/ml of bLf showed anti-HBV-DNA effect in HepG2 cells (*P* = 0.039, *n* = 3), however, 1.0, 0.5 and 0.1 mg/ml concentration of bLf could not inhibit HBV-DNA in HepG2 cells (*P* > 0.05, *n* = 3). 1.5, 1.0, and 0.5 mg/ml concentration of holo-bLf could not significantly inhibit HBV-DNA (*P* > 0.05, *n* = 3) except 0.1 mg/ml of holo-bLf showed marginal inhibitory effect on HBV-DNA (*P* = 0.048, *n* = 3). Any sub-group of apo-bLf could not inhibit HBV-DNA. In order to justify whether single Fe^3+^ has the inhibitory effect on HBV, FeCl_3_was involved in this anti-HBV tested. In this study, IFN-alpha 2B was used as negative control medicine and showed inhibitory effect on HBV-DNA (*P* = 0.001, *n* = 3), whereas FeCl_3_ could not inhibit HBV-DNA (*P* = 0.843, *n* = 3) (Table S3). Multiple comparison detail results were shown in Tables S4–S6. In addition, there are correlations between the anti-HBV effect of bLf, holo-bLf and its concentrations (*R* =  − 0.76, *P* = 0.0042, *R* = 0.86, *P* = 0.0003, respectively), and the relationship between the concentration of apo-bLf and the anti-HBV effect of apo-bLf was not significant (*R* = 0.36, *P* = 0.25) (Fig. S1).

## Discussion

In this study, iron-deficient and saturated bLf were prepared by bLf. Electrophoresis results showed that the candidate proteins were not degraded. Fluorescence spectrum of candidate proteins show that ions can bind to proteins, and with the increase of ions saturation, the fluorescence density decreases gradually and no other new peaks appear. These results indicate that the candidate protein can be used in the follow-up antiviral experiments. In this study, FQ-PCR was used to absolutely quantify the copy number of virus gene. The TC_0_ of bLf was 1.997 mg/ml, so we selected 1.5, 1.0, 0.5 and 0.1 mg/ml bLf in anti-HBV experiments. In the light of our results, 1.5 mg/ml bLf could inhibit HBV-DNA (*P* = 0.039, *n* = 3), and 1.0, 0.5 and 0.1 mg/ml bLf could not inhibit HBV-DNA in HepG2 cells (*P* > 0.05, *n* = 3). In Hara et al. (2002), nested polymerase chain reaction method was adopted to qualitative analysis of anti-HBV effect. The result showed that pretreating HBV serum with 1.0 or 0.1 mg/ml (higher concentration such as 1.5 mg/ml is not included) of bLf does not effectively inhibit HBV to infect PH5CH8 cells. This is basically consistent with our results. We performed a dose–response relationship analysis although only 1.5 mg/ml bLf was effective. The results showed that the concentration was negatively correlated with the log value of HBV copy number. This suggests that higher concentrations of the bLf may have better anti-HBV effects. It is necessary to carry out further antiviral experiments.

In our study, all concentrations of apo-bLf did not show anti-HBV effects. The two globular lobes of Lf are folded as alpha-helical structures, beta-sheet structures, or random coil structures (Legrand et al., 2008). At pH 2.0, apo-Lf contains less alpha-helical structures and random coil structures. We speculate that the deficiency of iron can change the proportion of alpha-helical and beta-sheet structure of Lf, which may affect the anti-HBV activity of Lf as a result. The results of our study show that the anti-HBV effect of candidate bLf is different because of the different conformational states although the iron saturation in apo- and native bLf form is nearly identical (8.42% and 10.29%). BLf could bound to iron at even pH 3.5 condition. However, apo-bLf requires more lower pH to maintain iron deficiency (Baker & Baker, 2012). Therefore, in the antiviral experiment of apo-bLf, the pH value in the solution will affect its anti-HBV effect. In addition, Lf exhibits a high positive charge with an isoelectric point of 9–10 and uneven surface charge distribution. The higher concentrations of Lf may cause the positive charge to change and favor adsorption to the cell membrane for bLf. This may explain why lower concentrations of bLf have no inhibitory effect.

In our study, 1.5, 1.0, 0.5 mg/ml holo-bLf has no effect on HBV-DNA, and marginal inhibitory effect was showed only in 0.1 mg/ml concentrations. Based on its binding metal status, Lf could express another conformational state. In the iron-binding role, Lf behaves as a classic binding protein. Each lobe of Lf is fully closed over and bound to one Fe^3+^ ion, each Fe^3+^ ion associated with a corresponding carbonate ion. This closed, metal-bound form is highly stable and relatively rigid as metal ions lock the domains together. Such a conformational change might explain why Lf has a low affinity for its receptors. A previous study has shown that iron-saturated bLf lose their ability to inhibit HSV (Marchetti et al., 1998). High concentration of holo-bLf may form aggregates or gels, and it may affect its adsorption capacity. We speculate that this closed, metal-bound form may also affect physical forces, such as hydrophobic interaction, hydrogen bonding and electrostatic interaction, at high concentrations. Interestingly, holo-bLf may be dissolved at low concentration, thus showing marginal anti-HBV effects. This may be the reason why low concentration of holo-bLf has anti-HBV effects. It is necessary to increase the sample size to evaluate the physiological significance of iron-saturated bLf in further study. In order to determine whether Fe^3+^ alone will affect the anti-HBV effect, 6 µg/ml of FeCl_3_ (which is almost equivalent to bLf with iron saturation of 1.5 mg/ml) was used in this antivirus study. The experimental results showed that iron ion itself could not inhibit HBV virus. Based on the results of this study, we believe that the anti-hepatitis B virus effect of bLf is related to its binding iron saturation.

There were studies demonstrating that bLf exhibit the anti-HBV ability after pre-incubated with cells suggesting an interaction with cell–surface receptors involved in viral adhesion (Hara et al., 2002; Li et al., 2009). Florian et al. (2013) reported that human Lf-derived synthetic peptide HLP_1−23_ could effectively prevent HBV to infect HepaRG cells, and also found that the major adhesion target is plasma membrane molecules of cell. It is known that Lf functions possibly by interacting with heparin sulfate and many other glycosaminoglycans. There is a study reporting that the intact Lf can significantly inhibit HSV-1 by competing for heparin sulphate proteoglycans at cell receptor binding sites (Marchetti et al., 1996). McAbee, Bennatt & Ling (1998) observed that Lf could bind to the asialoglycoprotein receptor in rat hepatocytes, which may be associated with HBV infection (Treichel et al., 1997). Our research shows that 1.5 mg/ml bLf can inhibit the replication of HBV after cells are infected with HBV. The relevant mechanism is not clear, and further study is needed.

The results of a pharmacokinetic experiment of Lf in rats and mice demonstrated that the liver could quickly clear the Lf from the bloodstream, and both hepatocytes and Kupffer cells had the ability to adhere to Lf (van der Strate et al., 2001). Another research revealed that human Lf distribution in the liver of patients affected by HBV is uneven, and the liver may intake Lf from the extracellular environment (Tuccari et al., 2002). Lf is relatively resistant to intestinal proteolytic enzymes, and the gastric survival of Lf is above 50% (Jiang & Lonnerdal, 2014; Troost et al., 2001). It can reach the small intestine, and undigested fragments of Lf in the urine and feces of infants have been found (Hamosh, 1998). Currently, there have been enteral interventions involving bLf (The ELFIN trial investigators group, 2019). Therefore, it is possible to test the anti-HBV activity of bLf *in vivo*; we will perform a controlled experimentation in animal models to confirm this hypothesis in a further study.

There were several limitations in this study. First, the anti-HBV mechanism of bLf under different percentages of iron saturation was not studied. Second, we did not conduct an antiviral intervention experiment in animals or humans with the protein. We will carry out further research in a future study. Third, our study observed the initial phase of the hepatitis B virus infection, long-term efficacy of bLf on HBV will be tested in a following experiment.

## Conclusion

A total of 1.5 mg/ml bLf and 0.1 mg/ml holo-bLf could inhibit HBV-DNA in HepG2 cells. Complete bLf structure, appropriate concentration and iron saturation of bLf are necessary conditions for anti-HBV effects.

## Supplemental Information

10.7717/peerj.17302/supp-1 Supplemental Information 1 Raw data

10.7717/peerj.17302/supp-2 Supplemental Information 2 Supplementary Data

## Additional Information and Declarations

### Data Availability

The following information was supplied regarding data availability: The raw data are available in the Supplemental Files.
