# Supplementary material for: Effect of iron saturation of bovine lactoferrin on the inhibition of hepatitis B virus in vitro
Source: PeerJ. 2024 May 7;12:e17302. doi: 10.7717/peerj.17302 (PMC11086297; doi:10.7717/peerj.17302)
Supplement: Supplemental Information 2 [file peerj-12-17302-s002.docx]

**Table S1** Test for normality of variance

|  | Kolmogorov-Smirnov | | | Shapiro-Wilk | | |
| --- | --- | --- | --- | --- | --- | --- |
|  | Statistic | df | Sig. | Statistic | df | Sig. |
| Lf | 0.273 | 18 | 0.001 | 0.771 | 18 | 0.001 |
| apo-bLf | 0.305 | 18 | 0.000 | 0.812 | 18 | 0.002 |
| holo-bLf | 0.239 | 18 | 0.008 | 0.824 | 18 | 0.003 |

**Table S2 Test for** **homogeneity of variance**

|  | Levin statistics | *v1* | *v2* | Sig. |
| --- | --- | --- | --- | --- |
| bLf | 1.198 | 5 | 12 | 0.367 |
| apo-bLf | 4.115 | 5 | 12 | 0.021 |
| holo-bLf | 3.041 | 5 | 12 | 0.053 |

**Table S3** Effect of Fe^3+^ inhibits HBV

| Multiple comparison | | Mean difference | Standard Error | Sig. | 95%CI | |
| --- | --- | --- | --- | --- | --- | --- |
|  |  |  |  |  | Lower | Upper |
| Positive | Fe^3+^ | 0.073 | 0.088 | 0.843 | -0.303 | 0.448 |

6 μg/ml FeCl_3_ were involved in this test. The infected cells without any treatment are defined as positive groups. * *P*<0.05

**Table S4** Multiple comparison of antiviral effect of bLf

| Multiple comparison | | Mean difference | Standard Error | Sig. | 95%CI | |
| --- | --- | --- | --- | --- | --- | --- |
|  |  |  |  |  | Lower | Lower |
| Positive | IFN-alpha 2B | 5.092* | 0.095 | 0.000 | 4.467 | 5.718 |
|  | bLf(1.5 mg/ml) | 5.129* | 0.309 | 0.039 | 0.586 | 9.673 |
|  | bLf(1.0 mg/ml) | 0.580 | 0.283 | 0.935 | -3.483 | 4.643 |
|  | bLf(0.5 mg/ml) | -0.072 | 0.333 | 1.000 | -5.072 | 4.928 |
|  | bLf(0.1 mg/ml) | 0.741 | 0.302 | 0.865 | -3.678 | 5.159 |
| IFN-alpha 2B | Positive | -5.092* | 0.095 | 0.000 | -5.718 | -4.467 |
|  | bLf(1.5 mg/ml) | 0.037 | 0.312 | 1.000 | -4.172 | 4.246 |
|  | bLf(1.0 mg/ml) | -4.512* | 0.287 | 0.033 | -8.236 | -0.789 |
|  | bLf(0.5 mg/ml) | -5.164 | 0.336 | 0.040 | -9.837 | -0.492 |
|  | bLf(0.1 mg/ml) | -4.352* | 0.306 | 0.043 | -8.434 | -0.269 |
| bLf(1.5 mg/ml) | Positive | -5.129* | 0.309 | 0.039 | -9.673 | -0.586 |
|  | IFN-alpha 2B | -0.037 | 0.312 | 1.000 | -4.246 | 4.172 |
|  | bLf(1.0 mg/ml) | -4.549* | 0.411 | 0.006 | -7.121 | -1.978 |
|  | bLf(0.5 mg/ml) | -5.201* | 0.447 | 0.005 | -7.992 | -2.411 |
|  | bLf(0.1 mg/ml) | -4.389* | 0.424 | 0.007 | -7.025 | -1.752 |
| bLf(1.0 mg/ml) | Positive | -0.580 | 0.283 | 0.935 | -4.643 | 3.483 |
|  | IFN-alpha 2B | 4.512* | 0.287 | 0.033 | 0.789 | 8.236 |
|  | bLf(1.5 mg/ml) | 4.549* | 0.411 | 0.006 | 1.978 | 7.121 |
|  | bLf(0.5 mg/ml) | -0.652 | 0.429 | 0.968 | -3.387 | 2.084 |
|  | bLf(0.1 mg/ml) | 0.161 | 0.406 | 1.000 | -2.372 | 2.694 |
| bLf(0.5 mg/ml) | Positive | 0.072 | 0.333 | 1.000 | -4.928 | 5.072 |
|  | IFN-alpha 2B | 5.164* | 0.336 | 0.040 | 0.492 | 9.837 |
|  | bLf(1.5 mg/ml) | 5.201* | 0.447 | 0.005 | 2.411 | 7.992 |
|  | bLf(1.0 mg/ml) | 0.652 | 0.429 | 0.968 | -2.084 | 3.387 |
|  | bLf(0.1 mg/ml) | 0.813 | 0.442 | 0.897 | -1.959 | 3.584 |
| bLf(0.1 mg/ml) | Positive | -0.741 | 0.302 | 0.865 | -5.159 | 3.678 |
|  | IFN-alpha 2B | 4.352* | 0.306 | 0.043 | 0.269 | 8.434 |
|  | bLf(1.5 mg/ml) | 4.389 | 0.424 | 0.007 | 1.752 | 7.025 |
|  | bLf(1.0 mg/ml) | -0.161 | 0.406 | 1.000 | -2.694 | 2.372 |
|  | bLf(0.5 mg/ml) | -0.813 | 0.442 | 0.897 | -3.584 | 1.959 |

Non-toxic concentrations of bLf are 1.5, 1.0, 0.5 and 0.1 mg/ml, respectively. 2,500 IU/ml IFN-alpha 2B were involved in this test. The infected cells without any treatment are defined as positive groups. * *P*<0.05

**Table S5** Multiple comparison of antiviral effect of apo-bLf

| Multiple comparison | | Mean difference | Standard Error | Sig. | 95%CI | |
| --- | --- | --- | --- | --- | --- | --- |
|  |  |  |  |  | Lower | Upper |
| Positive | IFN-alpha 2B | 3.380 | 0.377 | 0.067 | -0.425 | 7.185 |
|  | Apo-bLf(1.5 mg/ml) | -0.143 | 0.384 | 1.000 | -4.069 | 3.783 |
|  | Apo-bLf(1.0 mg/ml) | 0.527 | 0.160 | 0.524 | -0.896 | 1.949 |
|  | Apo-bLf(0.5 mg/ml) | 0.940 | 0.886 | 0.999 | -12.584 | 14.464 |
|  | Apo-bLf(0.1 mg/ml) | 0.517 | 0.204 | 0.632 | -0.751 | 1.784 |
| IFN-alpha 2B | Positive | -3.380 | 0.377 | 0.067 | -7.185 | 0.425 |
|  | Apo-bLf(1.5 mg/ml) | -3.523* | 0.498 | 0.031 | -6.622 | -0.425 |
|  | Apo-bLf(1.0 mg/ml) | -2.853 | 0.356 | 0.166 | -8.015 | 2.308 |
|  | Apo-bLf(0.5 mg/ml) | -2.440 | 0.941 | 0.768 | -12.151 | 7.271 |
|  | Apo-bLf(0.1mg/ml) | -2.863 | 0.378 | 0.101 | -6.655 | 0.928 |
| Apo-bLf(1.5 mg/ml) | Positive | 0.143 | 0.384 | 1.000 | -3.783 | 4.069 |
|  | IFN-alpha 2B | 3.523* | 0.498 | 0.031 | 0.425 | 6.622 |
|  | Apo-bLf(1.0 mg/ml) | 0.670 | 0.363 | 0.963 | -4.622 | 5.962 |
|  | Apo-bLf(0.5 mg/ml) | 1.083 | 0.944 | 0.998 | -8.519 | 10.686 |
|  | Apo-bLf(0.1mg/ml) | 0.660 | 0.384 | 0.962 | -3.252 | 4.572 |
| Apo-bLf(1.0 mg/ml) | Positive | -0.527 | 0.160 | 0.524 | -1.949 | 0.896 |
|  | IFN-alpha 2B | 2.853 | 0.356 | 0.166 | -2.308 | 8.015 |
|  | Apo-bLf(1.5 mg/ml) | -0.670 | 0.363 | 0.963 | -5.962 | 4.622 |
|  | Apo-bLf(0.5 mg/ml) | 0.413 | 0.877 | 1.000 | -14.149 | 14.975 |
|  | Apo-bLf(0.1mg/ml) | -0.010 | 0.161 | 1.000 | -1.446 | 1.426 |
| Apo-bLf(0.5 mg/ml) | Positive | -0.940 | 0.886 | 0.999 | -14.464 | 12.584 |
|  | IFN-alpha 2B | 2.440 | 0.941 | 0.768 | -7.271 | 12.151 |
|  | Apo-bLf(1.5 mg/ml) | -1.083 | 0.944 | 0.998 | -10.686 | 8.519 |
|  | Apo-bLf(1.0 mg/ml) | -0.413 | 0.877 | 1.000 | -14.975 | 14.149 |
|  | Apo-bLf(0.1mg/ml) | -0.423 | 0.886 | 1.000 | -13.932 | 13.085 |
| Apo-bLf(0.1mg/ml) | Positive | -0.517 | 0.204 | 0.632 | -1.784 | 0.751 |
|  | IFN-alpha 2B | 2.863 | 0.378 | 0.101 | -0.928 | 6.655 |
|  | Apo-bLf(1.5 mg/ml) | -0.660 | 0.384 | 0.962 | -4.572 | 3.252 |
|  | Apo-bLf(1.0 mg/ml) | 0.010 | 0.161 | 1.000 | -1.426 | 1.446 |
|  | Apo-bLf(0.5 mg/ml) | 0.423 | 0.886 | 1.000 | -13.085 | 13.932 |

Non-toxic concentrations of apo-bLf are 1.5, 1.0, 0.5 and 0.1 mg/ml, respectively. 2,500 IU/ml IFN-alpha 2B were involved in this test. The infected cells without any treatment are defined as positive groups. * *P*<0.05

**Table S6** Multiple comparison of antiviral effect of holo-bLf

| Multiple comparison | | Mean difference | Standard Error | Sig. | 95%CI | |
| --- | --- | --- | --- | --- | --- | --- |
|  |  |  |  |  | Lower | Upper |
| Positive | IFN-alpha 2B | 3.379 | 0.375 | 0.065 | -0.376 | 7.133 |
|  | holo-bLf(1.5 mg/ml) | -0.010 | 0.216 | 1.000 | -1.368 | 1.348 |
|  | holo-bLf(1.0 mg/ml) | 0.283 | 0.214 | 0.988 | -1.056 | 1.622 |
|  | holo-bLf(0.5 mg/ml) | 0.941 | 0.175 | 0.115 | -0.284 | 2.166 |
|  | holo-bLf(0.1 mg/ml) | 3.468* | 0.357 | 0.048 | 0.043 | 6.894 |
| IFN-alpha 2B | Positive | -3.379 | 0.375 | 0.065 | -7.133 | 0.376 |
|  | holo-bLf(1.5 mg/ml) | -3.389 | 0.382 | 0.056 | -6.913 | 0.136 |
|  | holo-bLf(1.0 mg/ml) | -3.095 | 0.381 | 0.073 | -6.659 | 0.468 |
|  | holo-bLf(0.5 mg/ml) | -2.438 | 0.360 | 0.192 | -6.994 | 2.118 |
|  | holo-bLf(0.1 mg/ml) | 0.090 | 0.476 | 1.000 | -2.878 | 3.058 |
| holo-bLf(1.5 mg/ml) | Positive | 0.010 | 0.216 | 1.000 | -1.348 | 1.368 |
|  | IFN-alpha 2B | 3.389 | 0.382 | 0.056 | -0.136 | 6.913 |
|  | holo-bLf(1.0 mg/ml) | 0.293 | 0.226 | 0.990 | -1.109 | 1.695 |
|  | holo-bLf(0.5 mg/ml) | 0.951 | 0.189 | 0.163 | -0.469 | 2.370 |
|  | holo-bLf(0.1 mg/ml) | 3.478* | 0.364 | 0.040 | 0.261 | 6.695 |
| holo-bLf(1.0 mg/ml) | Positive | -0.283 | 0.214 | 0.988 | -1.622 | 1.056 |
|  | IFN-alpha 2B | 3.095 | 0.381 | 0.073 | -0.468 | 6.659 |
|  | holo-bLf(1.5 mg/ml) | -0.293 | 0.226 | 0.990 | -1.695 | 1.109 |
|  | holo-bLf(0.5 mg/ml) | 0.657 | 0.186 | 0.389 | -0.725 | 2.040 |
|  | holo-bLf(0.1 mg/ml) | 3.185 | 0.363 | 0.053 | -0.066 | 6.437 |
| holo-bLf(0.5 mg/ml) | Positive | -0.941 | 0.175 | 0.115 | -2.166 | 0.284 |
|  | IFN-alpha 2B | 2.438 | 0.360 | 0.192 | -2.118 | 6.994 |
|  | holo-bLf(1.5 mg/ml) | -0.951 | 0.189 | 0.163 | -2.370 | 0.469 |
|  | holo-bLf(1.0 mg/ml) | -0.657 | 0.186 | 0.389 | -2.040 | 0.725 |
|  | holo-bLf(0.1 mg/ml) | 2.528 | 0.341 | 0.153 | -1.662 | 6.717 |
| holo-bLf(0.1 mg/ml) | Positive | -3.468* | 0.357 | 0.048 | -6.894 | -0.043 |
|  | IFN-alpha 2B | -0.090 | 0.476 | 1.000 | -3.058 | 2.878 |
|  | holo-bLf(1.5 mg/ml) | -3.478* | 0.364 | 0.040 | -6.695 | -0.261 |
|  | holo-bLf(1.0 mg/ml) | -3.185 | 0.363 | 0.053 | -6.437 | 0.066 |
|  | holo-bLf(0.5 mg/ml) | -2.528 | 0.341 | 0.153 | -6.717 | 1.662 |

Non-toxic concentrations of holo-bLf are 1.5, 1.0, 0.5 and 0.1 mg/ml, respectively. 2,500 IU/ml IFN-alpha 2B were involved in this test. The infected cells without any treatment are defined as positive groups. * *P*<0.05

**
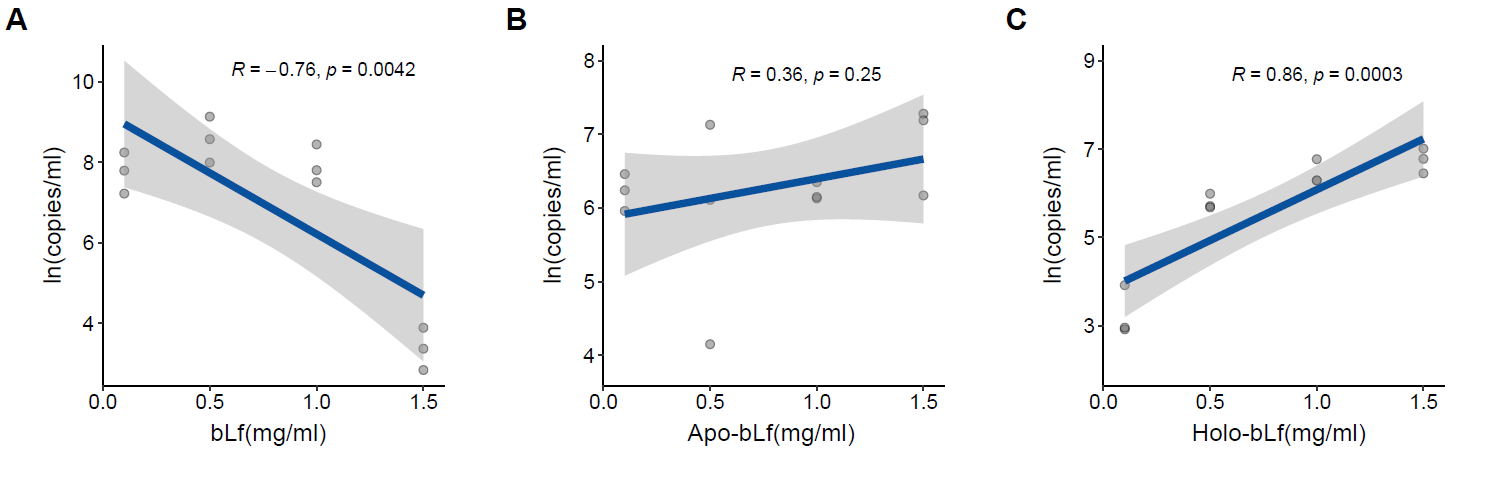
**

**Figure S1** Correlation analysis between anti-HBV effect of candidate proteins and protein concentrations. A: Relationship between the anti-HBV effect of bLf and its concentrations. B: Relationship between the anti-HBV effect of apo-bLf and its concentrations. C: Relationship between the anti-HBV effect of holo-bLf and its concentrations.
